# Assessment of gentamicin and cisplatin-induced kidney damage mediated via necrotic and apoptosis genes in albino rats

**DOI:** 10.1186/s12917-021-03023-4

**Published:** 2021-11-16

**Authors:** Tarek Kamal Abouzed, Eman Abd Elrahman Sherif, Mohamed El Sayed Barakat, Kadry Mohamed Sadek, Adil Aldhahrani, Nasr Elsayed Nasr, Ehab Eldomany, Khaled Khailo, Doaa Abdallha Dorghamm

**Affiliations:** 1 Biochemistry Department, Faculty of Veterinary Medicine Kafrelsheikh University, Kafrelsheikh, Egypt; 2 Biochemistry Unit, Animal Health Research Institute, Kafrelsheikh branch. Agricultural Research Center (ARC), Kafrelsheikh, Egypt; 3 Biochemistry Department, Faculty of Veterinary Medicine Damanhour University, Damanhour, Egypt; 4 Clinical laboratory science Department, Turabah University College, Taif University, Taif, Saudi Arabia; 5 Department of Biotechnology and Life science, Faculty of Postgraduate Studies for Advanced Science Beni-suef University, Beni-suef, Egypt

**Keywords:** *Gentamycin*, *Cisplatin*, Nephrotoxicity, TNFα, Caspase 3, Bax, BCL2 genes

## Abstract

**Background:**

Gentamicin (GM) is a low-cost, low-resistance antibiotic commonly used to treat gram-negative bacterial diseases. Cisplatin (Csp) is a platinum-derived anti-neoplastic agent. This experiment aimed to identify the early signs of gentamicin and cisplatin-induced nephrotoxicity in rats. Thirty *Wistar* rats were divided into three groups of 10: a control group, which received no treatment; a gentamicin group administered by a dose of (100 mg/kg, IP) for 7 consecutive days, and a cisplatin group was administered intraperitoneal in a dose of (1.5 mg/kg body weight) repeated twice a week for 3 weeks.

**Results:**

Both experimental groups exhibited increased levels of creatinine, urea, and uric acid, with the cisplatin-treated group showing higher levels than the gentamicin group. Experimental groups also exhibited significantly increased Malondialdehyde (MDA), reduced glutathione (GSH), and glutathione peroxidase (GSH-Px) with more pronounced effects in the cisplatin-treated group. Further, both experimental groups exhibited significant up-regulation of Tumor Necrosis Factor α (TNF-α), caspase-3, and Bax and down regulation of Bcl-2.

**Conclusion:**

These findings confirm the use of necrotic, apoptotic genes as early biomarkers in the detection of tubular kidney damage. Further, cisplatin was shown to have a greater nephrotoxic effect than gentamicin; therefore, its use should be constrained accordingly when co-administered with gentamicin.

## Background

The kidneys have a role within some key functions around homeostasis and detoxification, including the excretion of toxic metabolites and some medications [1]. As such, they play an important role in processing toxic drugs and are consequently more exposed to harmful substances via high renal blood flow, which transports metabolites and picks up toxic chemicals from the surrounding fluid [2]. Pharmacological interventions such as interleukin-2, Gentamicin, Ibuprofen, Vancomycin, Furosemide, and chemotherapeutic treatments containing cisplatin, carboplatin, and mitomycin, can have nephrotoxic effects [3].

The aminoglycoside, Gentamicin (GM) is a low-cost, low-resistance antibiotic commonly used to treat gram-negative bacterial diseases [4]. However, its nephrotoxicity and ototoxicity are significant factors leading to constraint in the use of aminoglycosides in general [5]. Gentamicin has the following nephrotoxic effects: 1) accumulation in the proximal convoluted tubule [6], which triggers 2) tubular necrosis and glomerular congestion, leading to glomerular and renal dysfunction [7]. Furthermore, it causes oxidative stress and inflammatory cascades, which are significant nephrotoxic factors [8].

Cisplatin or *cis-diamminedichloroplatinum II* (Csp) is a platinum-derived anti-neoplastic agent, commonly used in the treatment of carcinoma, lymphoma, and germ-cell tumors [9]. Despite being an efficient chemotherapeutic drug, cisplatin’s nephrotoxicity limits its long-term use [10]. Consequently, it is mainly excreted by the kidneys [11] and accumulates in mitochondria, causing changes in bioenergetics [12]. Furthermore, cisplatin has been implicated in oxidative stress-induced kidney damage [13] as well as apoptosis of kidney tubular cells – although the mechanism for this is not well understood. Cisplatin’s toxicity is thought to be mainly derived from DNA damage; prevention of protein formation; and damage to mitochondria, causing apoptosis [14]. Apoptosis is a significant cause of inflammation and implicated in several diseases of the kidney associated with nephrotoxic drugs [15, 16].

Apoptotic renal damage caused by Gentamicin and cisplatin is heavily dependent on caspase-based signaling. Caspase-9 triggers the release of caspase-3 within the mitochondrial pathway [17]. The proteins Bax and Bcl-2 also play a significant role, with Bax serving a pro-apoptotic function and Bcl-2 as an anti-apoptotic. Specifically, Bcl-2 blocks cytochrome c activation by binding to the mitochondrial membrane [18]. Furthermore, nuclear translocation and the activation of TNF-α lead to oxidative stress and renal inflammation, which significantly impacts cytokine, chemokine, and adhesion molecule expression in the basal gene [19].

## Results

### Effects of *gentamicin* and *Cisplatin* on biochemical parameters

**Fig. 1:**
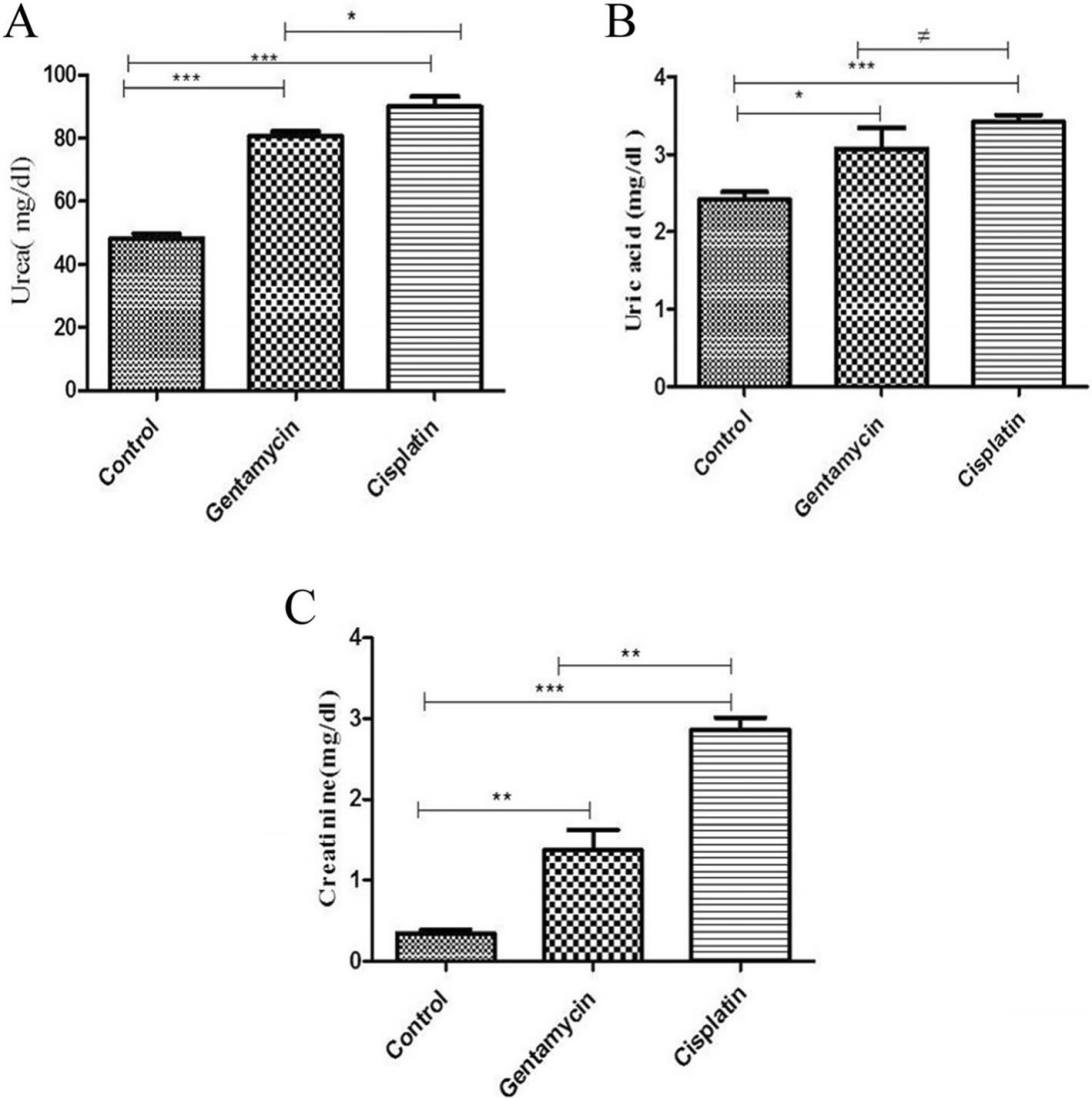
Effect of Gentamycin and cisplatin **a** Urea (mg/dl) **b** Uric acid (mg/dl), **c** creatinine (mg/dl) the result represent the means ± SEM *P < 0.05

Serum levels of creatinine, uric acid, and urea were significantly elevated in the gentamicin and cisplatin groups compared with the control group (Fig. 1), with cisplatin showing higher elevation.

### Effects of *gentamicin* and *Cisplatin* on kidney MDA, GSH, and GPx activities

**Fig. 2:**
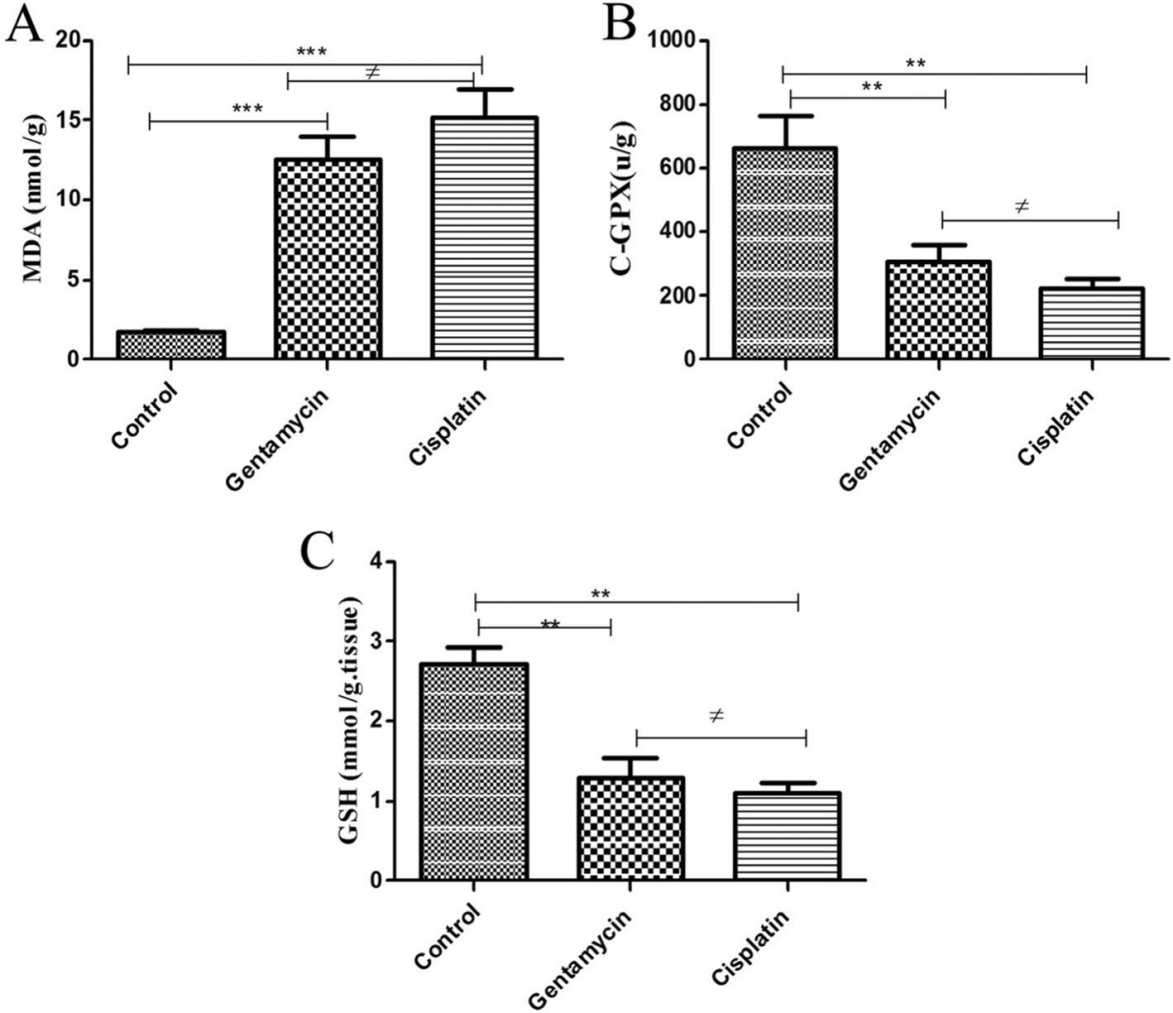
Effect of Gentamycin and cisplatin **a** MDA (nmol/g) **b** C-GPX (mg/dl), the result represent the means ± SEM *P < 0.05

MDA levels were significantly increase in the experimental groups compared with the control group, with the cisplatin-treated group showing the higher elevation. The cisplatin group also showed a significant decrease in GSH and GPx compared with the control group (Fig. 2).

### Histopathological analysis

**Fig. 3:**
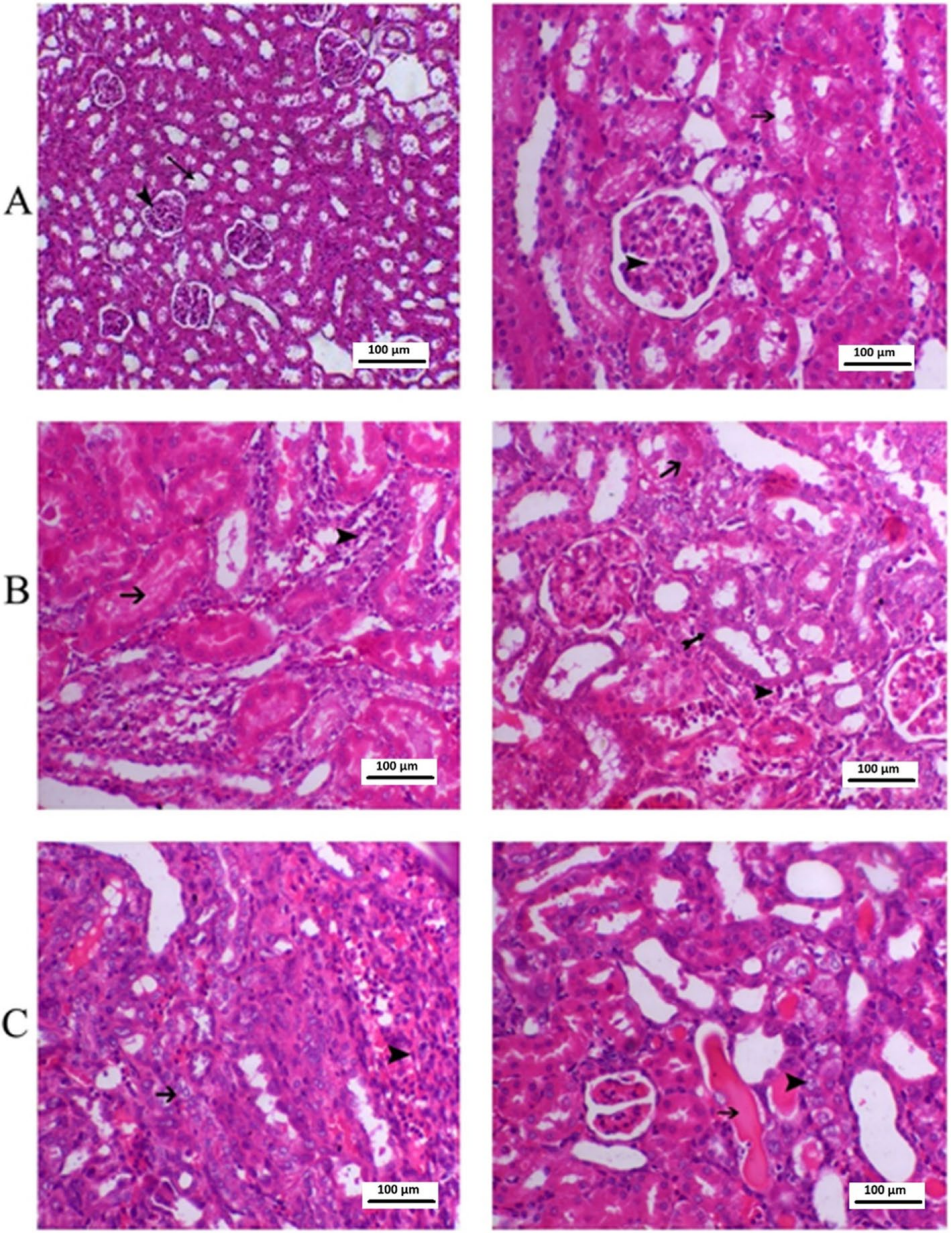
**A** Control (renal tubules are normal) **B**. Gentamycin group **C** cisplatin group. Many cortical convoluted tubules were revisited by necrotic epithelial cells (horizontal solid arrows) or vacuolated swell cells (arrow heads), glomeruli exhibited swelling with reduction of bowman’s capsular space. Shown are numerous inflammatory cells (small hollow vertical arrow) in the glomerular and tubular structures in the outer medulla

The control group exhibited normal renal glomerular and tubular structure in the cortical and medullary areas. The gentamicin group exhibited pathological abnormalities in the glomerular and tubular structures. Specifically, there was degeneration of the glomerular wall and some hypertrophy, as well as mononuclear cell infiltration, tubular epithelial cell degeneration, and inter-tubular hemorrhage (Fig. 3). The cisplatin group exhibited severe glomerular congestion, with infiltration of the inflammatory cells within the interstitium and severe hemorrhaging in the medullary and cortical areas. Furthermore, severe necrosis was observe in the tubule (Fig. 3).

### Effects of *gentamicin* and *cisplatin* on mRNA expression of TNF-α, Caspase-3, Bax, and Bcl-2

**Fig. 4:**
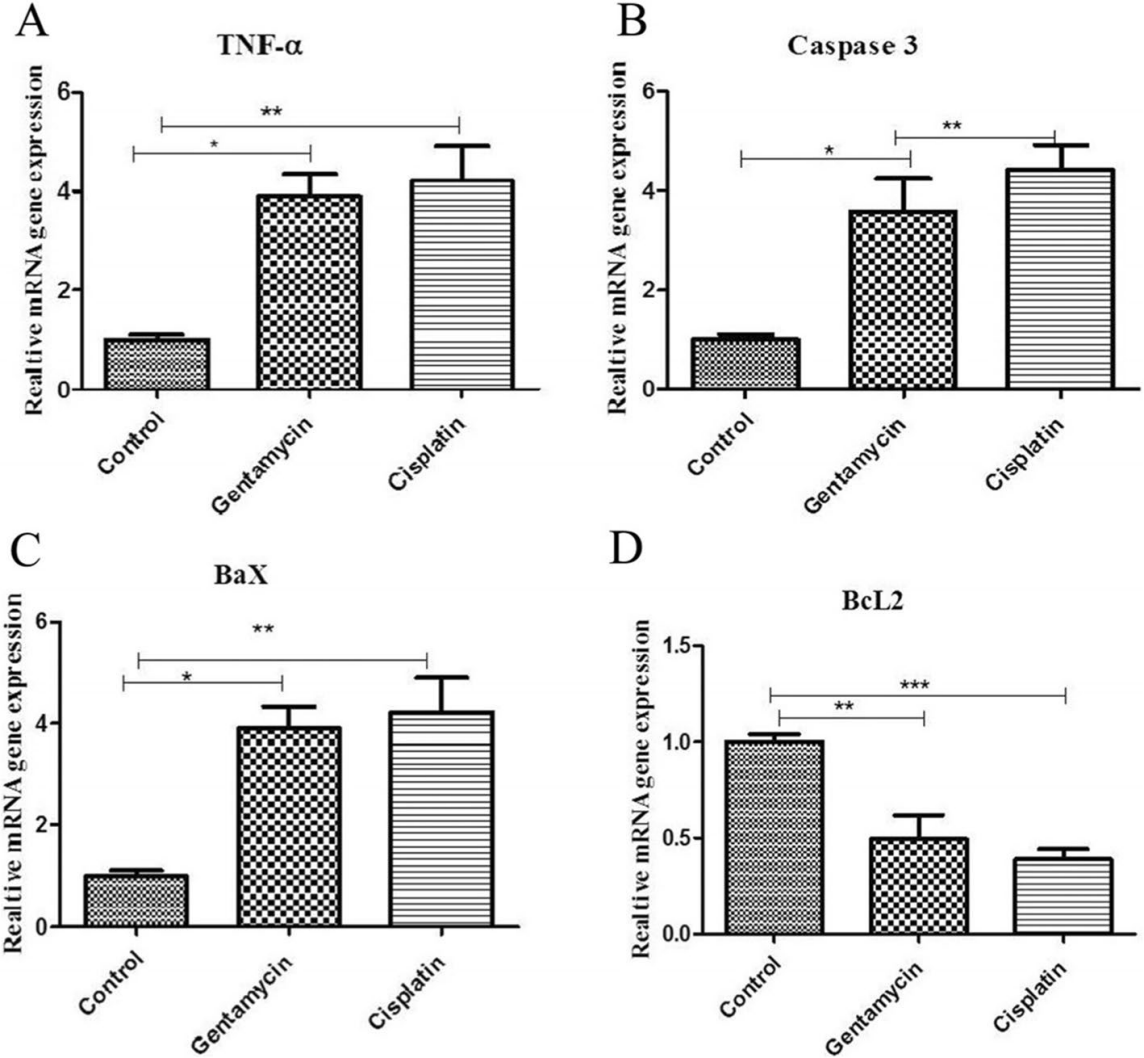
Effect of Gentamycin and cisplatin on mRNA expression **a** TNF-α **b** Caspase 3, (c) Bax and (D) BCL2 the result represent the means ± SEM *P < 0.05

TNF-α, Bax, and caspase-3 gene expression were significantly upregulated (*P* ≤ 0.05) in the cisplatin and gentamicin-administered rats. This was higher in the cisplatin group, as revealed by qPCR. Bcl-2 mRNA expression was downregulated in the two experimental groups, especially in the cisplatin group (Fig. 4).

## Discussion

Elevated serum creatinine and urea nitrogen levels can lead to renal dysfunction [20, 21]. In particular, increased serum levels of creatinine inhibits glomerular filtration [22], while high nitrogen levels in blood urea can indicate renal failure to cleanse urea [23]. This experiment found that administering gentamicin led to a significant increase in creatinine, urea, and uric acid in rats. These conform with findings by [24, 25], who also reported increased serum creatinine elevation and blood urea and uric acid, leading to the suggestion that gentamicin is nephrotoxic - although its exact mechanism is unclear. Some studies have implicated ROS formation caused by aminoglycoside antibiotics. Lipid peroxidation generates MDA in the tissues, which inhibits the amount of polyunsaturated fatty acids, which act as a substratum free radicals. This interaction, between phospholipids and aminoglycosides, is the first step in developing Gentamicin toxicity [26]. Moreover, Gentamicin forms an Iron-GEN complex, with iron liberated from the renal cortical mitochondria. This also triggers free radical formation and enhances ROS generation [27].

The increased MDA levels observed in this experiment aligns with the previous research [9, 28], indicating either elevated serum creatinine and urea levels, or elevated MDA in the kidney tissue. This suggests a link between lipid peroxidation and nephrotoxicity, oxidative stress, and kidney dysfunction.

Glutathione plays a critical role in cell maintenance. However, xenobiotics or peroxide-dependent changes in GSH tissue and antioxidant enzyme activity are a contentious topic at the moment [24]. This experiment observed that GSH levels in the kidney are inhibited by gentamicin, correlating with the findings of [29, 30]. These works found a link between gentamicin-induced nephrotoxicity, low GSH, and GSH-Px activity in the renal cortex, which might in turn lead to oxidative damage due to decreased antioxidant defenses. Inactivation of GSH-Px, CAT, and SOD would therefore fail to defend against the increased ROS levels produced by gentamicin. Gentamicin accumulates in the renal proximal convoluted tubules, degenerating the brush border membranes, generating free radicals, reducing antioxidant defenses, and causing glomerular congestion, acute tubular necrosis, and ultimately kidney failure [7, 31, 32]. Likewise, gentamicin administration causes renal oxidative damage due to antioxidant defense enzyme deficiencies [33, 34].

Tumor necrosis factor (TNF-α) is implicated in the pathogenesis of acute nephrotoxin-induced renal failure as well as other forms of kidney damage [35, 36]. The administration of gentamicin in our study caused TNF-α to increase significantly. These results are consistent with [7], who found that gentamicin-induced tubular injury caused inflammation at the site of the necrosis.

The pro-apoptotic gene, Bax, plays a key role, causing the release of cytochrome c, activating procaspase-9. This initiates various caspase streams including caspase-3 [37], which cleaves specific target proteins, causing the number of apoptotic cells to increase. Conversely, Bcl-2, an antiapoptotic protein, binds to the external membrane of mitochondria, blocking cytochrome c activation [18].

In this experiment, gentamicin upregulated TNF-α, Bax, and Caspase-3 mRNA expression, and downregulated Bcl-2 mRNA expression. This concurs with [38, 39], whose findings confirmed the same effects of gentamicin administration. From this, it can be conclude that gentamicin therapy can lead to inflammation and apoptosis in renal tissues, followed by necrosis.

The clinical effects of cisplatin are linked to dosage, but high doses can be both nephrotoxic and neurotoxic [40]. Concerning the renal system, cisplatin damages the proximal tubules [41].

This experiment found that cisplatin administered rats exhibited increased creatinine, urea, and uric acid and this effect was more pronounced than in rats treated with gentamicin, agreeing with the work by [42, 43]. Histopathological observations confirmed damage to the glomerular wall, epithelial degeneration, intertubular hemorrhage, and slight glomerular hypertrophy.

Cisplatin-generated ROS targets organelles, including two microsomes and the mitochondria [44, 45], causing apoptosis and necrosis [46, 47]. Some molecules are also targeted, such as lipids and proteins, causing an increase in MDA and a decrease in the antioxidants GSH and GPx [48, 49]. This work observed that rats administered with cisplatin exhibited increased tissue MDA and reduced kidney GSH, while the production of GSH-Px remained unchanged. These results agree with [50, 51], who recorded imbalanced antioxidant status caused by the buildup of excessive cisplatin-produced ROS. This led to depleted GSH and lipid peroxidation.

Apoptosis is central to many renal disorders, especially those that involve inflammatory processes or induced by nephrotoxic drugs [15, 16]. The present experiment found that cisplatin administration upregulated TNF-α, Bax, and caspase-3 mRNA expression, and downregulated Bcl-2 mRNA expression. These effects were more pronounced than in the gentamycin-administered group and agree with findings by [52, 53].

## Conclusion

This experiment confirmed the key pathogenic role played by ROS, TNF-α, and apoptotic proteins such as Bax, Bcl-2, and caspase-3 in gentamicin and cisplatin-induced nephrotoxicity. Furthermore, it found that of the two, cisplatin has the most damaging effect on the kidney.

## Materials and methods

### Chemicals

Gentamicin ampoules 80 mg (Alexandria Chemical Co., Egypt), creatinine kits (Diamond, Egypt), urea kits (Biomed, Egypt), uric acid kits (Spectrum, Egypt), malondialdehyde, glutathione reductase, and glutathione peroxidase (Biodiagnostics Co., Egypt), trizol (GENEzol™ RNA extraction reagent, Lot≠:30117B07003; Genetix Biotech Asia Pvt. Ltd., India), single-strand complementary DNA kit (cat.No.25014, iNtRON Biotechnology, South Korea), SYBR Green qPCR (cat≠RT500, Enzynomics, South Korea).

### Animals

This experiment was designed using ‘the ethical principles and guidelines for the care and use of laboratory animals’, and granted ethical approval by the Research Ethics Committee, Faculty of Veterinary Medicine, Kafrelsheikh University (Date: 13/1/2019). Thirteen adult male Wistar rats, each weighing 160-200 g, were obtained from faculty of Science lab, Kafrelsheikh University. The rats were kept at 25 °C on a 12/12 h light/dark cycle, in single plastic cages with bedding, with access to standard rat food and water ad libitum. They handled for 1 week before the study to adapt to their surroundings.

### Experimental design

Rats were randomly (ranking method) assigned to one of three groups (10 rats/group): 1) Control group, who received no intervention and maintained a regular diet; 2) Gentamicin group, who were administered 100 mg/kg BW IP gentamicin daily for 7 days [54, 55]; 3) Cisplatin group, who were administered 1.5 mg/kg BW IP twice a week for 3 weeks [56].

### Blood sample

Following the experimental phase, rats were anesthetized using 5% isoflurane in an induction chamber [57]. Following the loss of righting reflex, rats were rapidly transferred to a nose cone mask, and maintained with isoflurane with room air. Isoflurane anesthesia was performed using a rodent inhalant anesthesia apparatus (SomnoSuite Small Animal Anesthesia System, Kent Scientific Corporation, Connecticut). The flow rate of isoflurane was determined using following formula; Flow rate (ml/min) = 0.65 × body weight (g). and blood samples (6 samples/group) were collected from retro-orbital venous plexuses (7 ml blood/sample). The selection was not based on any pre-specified effect. The samples were centrifuged for 15 min at 3000 rpm in non-heparinized tubes. Sera were separated and stored at − 20 °C for later use. The investigators were not blinded during data collection. Blinding was used during analysis. Computational analysis was not performed blinded.

### Tissue sample

Rats were anesthetized with isoflurane and executed via cervical dislocation (euthanasia) and the kidneys were removed. The left kidneys were washed with liquid nitrogen then stored at − 80 °C for real-time assessment, while a portion was separated (1 g/sample) and stored at − 20 °C for MDA, GSH, and GSH-Px analysis. Each tissue sample (6 samples/group) was homogenized in 5 ml phosphate buffer pH 7.4 using an electrical homogenizer where the sample was maintained on ice. After homogenization, N-ethylmaleimide was added directly to prevent oxidation of GSH. Tissue homogenate was centrifuged at 1200×g for 20 min at 4 °C. The resulting supernatant was isolated and used in the assessment of the MDA, GSH, and GSH-Px in the renal tissue. The right kidneys were stored in 10% formalin for histopathological analysis. All rats and remnants of the samples were buried in the strict hygienically controlled properly constructed burial pit.

### Biochemical analysis

Colorimetric analysis was carried out on creatinine [58], urea [59], and uric acid [60], while calorimetric analysis of kidney homogenate measured malondialdehyde [61], reduced glutathione [62], and glutathione peroxidase [63].

### Histopathological analysis

Kidney tissue samples, previously stored in 10% neutral formalin, were paraffinised, sectioned, and stained with hematoxylin and eosin (H&E). The microscopy images captured by (The light microscope supplied by a digital camera computer device (Nikon digital camera; Japan) for examination of kidney section at resolution of 300 pixel.

### Quantitative determination of TNF-α, caspase-3, Bax, and Bcl-2 using real-time qPCR

Total RNA was isolated from kidney tissue using TRIzol, according to the manufacturer’s instructions. RNA concentration was measured using the Nanodrop spectrophotometer (Nanodrop 2000c, Thermos Scientific, USA), while single strand complementary DNA was synthesized using the HiSenScript™ cDNA synthesis kit. This involved mixing 10 μl 2X RT reaction buffer, 1 μl enzyme mix solution, and 1 μg RNA, then made up to 20 μl with RNase free water. This was incubated for 30 min at 50 °C then 10 min at 85 °C.

qPCR reactions were carried out using SYBR Green qPCR Master Mix and specific primers (see Tables 1 and 2). The following protocol was used: Initial denaturation for 10 min at 92 °C; 40 cycles at 92 °C for 15 s, 60 °C for 30s and 72 °C for 30s. The 2-△△Ct method [64] was used to estimate the differences in gene expression. This was standardized against β-actin and mRNA levels were recorded relative to the control. After amplification, the products were verified using a melting curve analysis.

**Table 1:** Sequences of primers used in qPCR

Gene	Accession no	Direction	Primer sequence
**Bcl2**	L14680	Sense	GACTTCGCCGAGATGTCCAG
		Antisense	GTGCAGGTGCCGGTTCAGG
**Bax**	U49729	Sense	AGGTCTTTTTCCGAGTGGCAGC
		Antisense	CCGGAGGAAGTCCAATGTCC
**Caspase 3**	GCA-00000189504	Sense	GGTATTGAGACAGACAGTGG
		Antisense	CATGGGATCTGTTTCTTTGC
**TNF-a**	GCA-00000189504	Sense	AAATGGGCTCCCTCTCATCAGTTC
		Antisense	TCTGCTTGGTGGTTTGCTACGAC
**β-actin**	V01217	Sense	GGACCTGACAGACTACC
		Antisense	GGCATAGAGGTCTTTACGG

**Table 2:** Effect of gentamicin and Body weight condition

	Control	Gentamicin	Cisplatin
Body weight	232.1 ± 6.25^a^	194.1 ± 8.52^b^	161.4 ± 7.75^c^
Kidney weight	0.596 ± 0.036^c^	0.732 ± 0.028^b^	0.840 ± 0.030^a^
Relative Kidney weight	0.0025 ± 0.0002^c^	0.0039 ± 0.0002^b^	0.0052 ± 0.0003^a^
Mortality rate	0%	10%	20%

Data are the mean ± SEM, with different small letter in the same row show significantly different at *p* < 0.05 using ANOVA followed by Tukey’s as a post-hoc test

### Statistical analysis

GraphPad Prism 5 (GraphPad Software, San Diego, USA) was used to conduct a one-way analysis of variance (ANOVA), followed by Tukey’s multiple comparisons post hoc test. *P* < 0.05 was considered statistically significant, with results expressed as means ± standard error (SE).

## Data Availability

The datasets used and/or analysed during the current study are available from the corresponding author on reasonable request.

## Abbreviations

GM: Gentamicin
Csp: Cisplatin,
I.P: Intraperitoneal
MDA: Malondialdehyde
GSH: Reduced glutathione
GSH-Px: Glutathione peroxidase
CAT: Catalase
SOD: Superoxide dismutase
ROS: Reactive oxygen species
DNA: Deoxyribonucleic acid
TNF-α: Tumor Necrosis Factor α
Bcl-2: B-cell lymphoma 2
